# A Rare Presentation of Extragenital Bowenoid Papulosis

**DOI:** 10.7759/cureus.24712

**Published:** 2022-05-03

**Authors:** Munir H Idriss, Jake Besch-Stokes, Spencer Bezalel, Leah Swanson, Julia S Lehman

**Affiliations:** 1 Dermatology, Mayo Clinic, Rochester, USA; 2 Dermatology, Mayo Clinic Alix School of Medicine, Phoenix, USA

**Keywords:** immunohistochemical staining, human papillomavirus (hpv), extragenital, facial, bowenoid papulosis

## Abstract

Bowenoid papulosis is an uncommon skin disorder usually seen in the genital area and associated with human papillomavirus (HPV) infection. Clinically, patients usually present with solitary or multiple skin- to brown-colored papules. Plaque morphology of lesions and extragenital location are unusual. Diagnosis is mainly based on clinical presentation and confirmed with a skin biopsy demonstrating keratinocyte atypia. Chromogen in situ hybridization for HPV can also be done. Herein, we present a rare case of bowenoid papulosis with a plaque morphology on the face with no concomitant involvement of the anogenital, oropharyngeal, or periungual areas in an immunocompromised patient. Histopathologic sections stained positive with the in situ hybridization technique for high-risk oncogenic HPV serotypes (16, 18, 31, 33, 35, 45, 51, 52, and 56), confirming the diagnosis.

## Introduction

Bowenoid papulosis is a human papillomavirus (HPV)-induced condition usually presenting as solitary or multiple skin- to brown-colored papules in the anogenital area. Isolated extragenital bowenoid papulosis is a rare phenomenon, described in a few cases following the first report by Bart in 1984 [1]. The majority of previous cases have occurred in the head and neck region with a papular morphology. However, other anatomic locations have been described [2-4].

## Case presentation

A 60-year-old male with a history of chronic immunosuppression (on mycophenolate mofetil and prednisone) after a renal transplant (2006) and pancreatic transplant (2007) presented to the dermatology clinic for a full skin examination. His past dermatologic history is remarkable for squamous cell carcinoma (SCC) in situ on the left shoulder treated with electrodesiccation and curettage several years ago. He reported a skin lesion over the right angle of his mouth and right chin of a few months’ duration. The lesion had been stable in size and otherwise asymptomatic. He denied any skin lesions over the anogenital or periungual areas or having oral sex. On review of systems, he did endorse intermittent difficulty swallowing.

**Figure 1:**
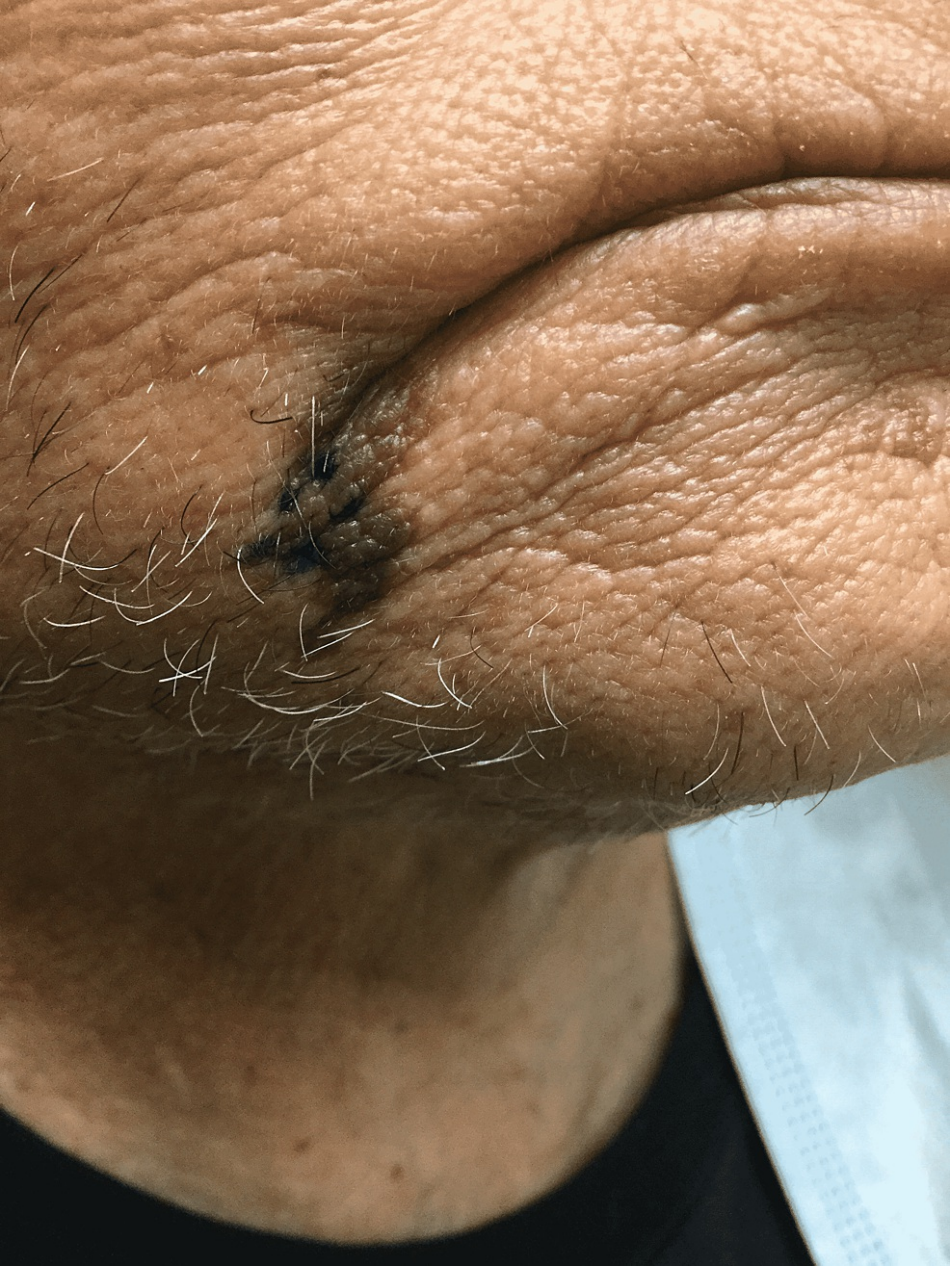
A thin tan to brown plaque located over the right chin.

**Figure 2:**
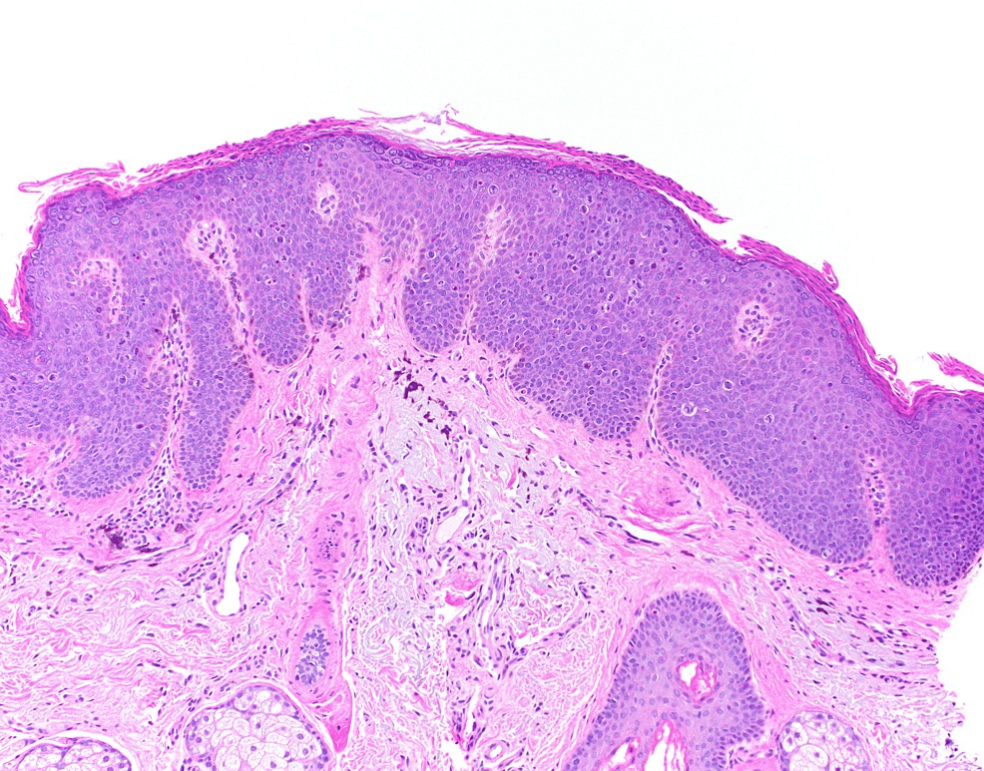
Histologic section demonstrating atypia of the epidermis with bowenoid features (hematoxylin and eosin stain, original magnification 100×).

**Figure 3:**
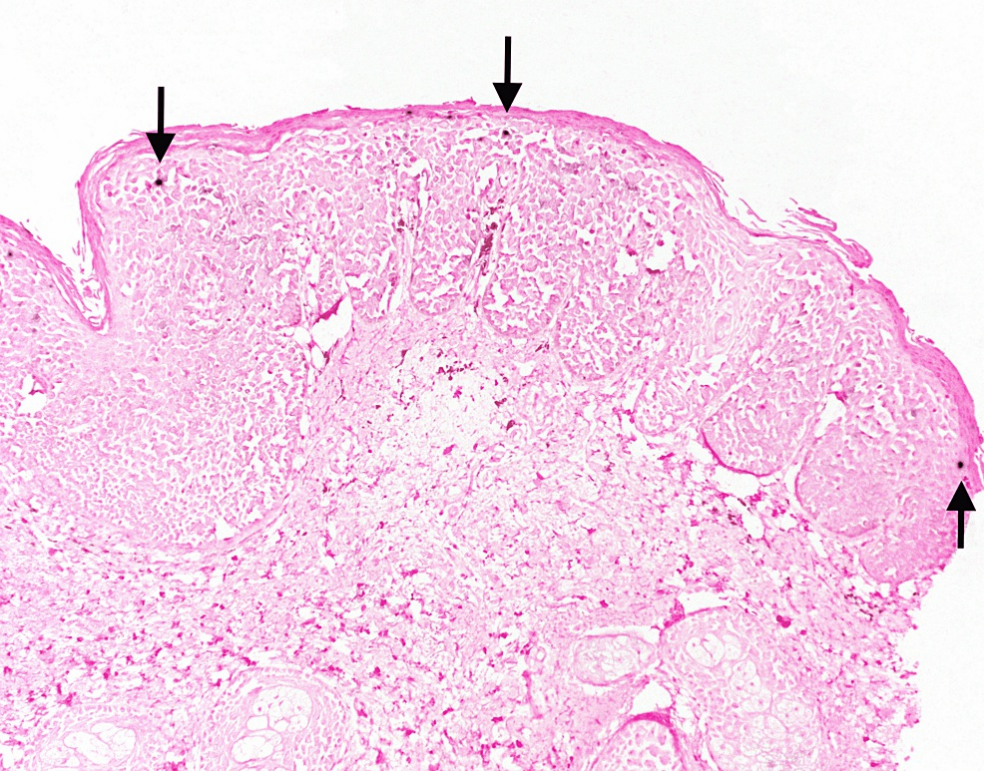
High-risk human papillomavirus (HPV) in situ hybridization staining demonstrating focal staining (black arrows) (original magnification 100×).

**Figure 4:**
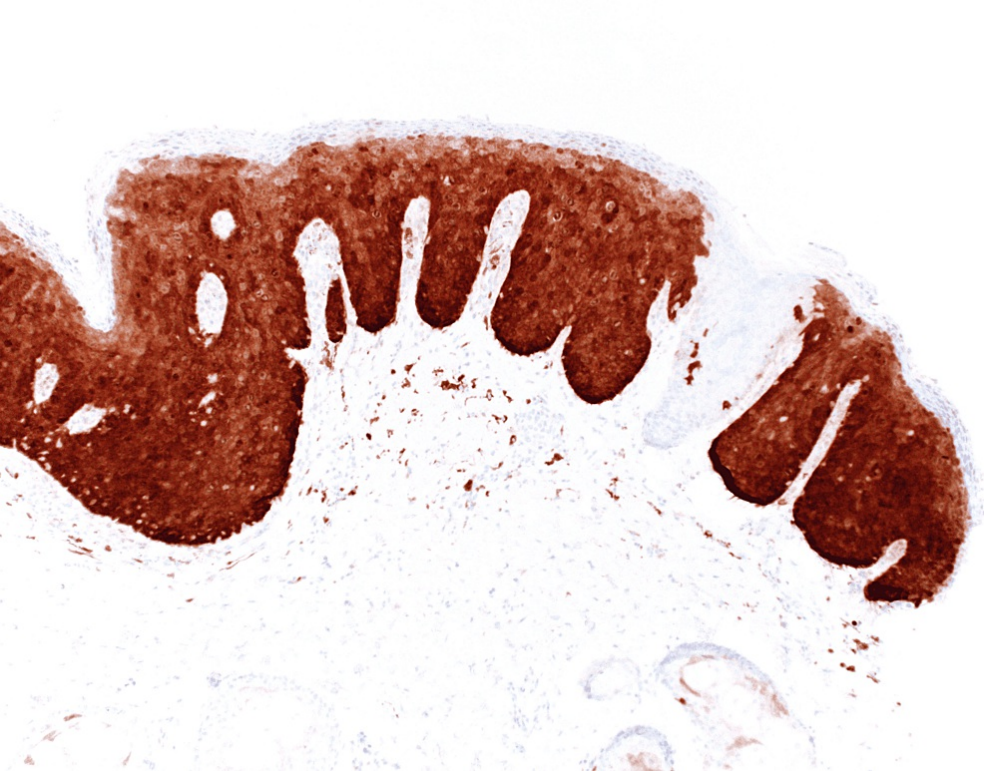
p16 immunohistochemical staining demonstrating diffuse staining (original magnification 100×).

Physical examination was remarkable for an irregularly bordered thin brown plaque extending from the right oral commissure to the right chin (Figure 1). The dermoscopic evaluation was remarkable for the reverse pigment network and focal areas of loss of pigment. There were no worrisome lesions over the genital and perianal areas. He did not have any palpable cervical lymphadenopathy. Due to the large size of the lesion, a punch biopsy was obtained from the clinically most atypical area. The biopsy was consistent with bowenoid papulosis (Figure 2). High-risk human papillomavirus (HPV) in situ hybridization was positive (Figure 3). p16 immunohistochemical staining was diffusely positive (Figure 4).

The patient was referred to ENT for evaluation of his intermittent difficulty swallowing and diagnosis of bowenoid papulosis. Oral examination and video laryngoscopy studies were negative. Due to the pathology and location, the patient was treated with Mohs micrographic surgery, and the tumor was cleared in one stage. The patient was lost to follow-up.

## Discussion

We present an interesting case of an isolated extragenital bowenoid papulosis of the face in a chronically immunosuppressed patient. Our patient’s lesion presented as a large thin plaque rather than classic papules, an unusual presentation [2,4]. Our case and previous reports highlight the varied clinical presentation of extragenital bowenoid papulosis and the need for a broad differential when evaluating unusually appearing, isolated papules or plaques, particularly of the head and neck.

In our case, the diagnosis was suggested by the bowenoid features seen on punch biopsy, as well as further positive high-risk HPV in situ hybridization and diffuse staining on p16 immunohistochemistry. Overall, the finding of bowenoid features on punch biopsy, even in a patient without concomitant anogenital involvement, should prompt further testing for high-risk HPV. Studies have demonstrated that staining for p16 has high sensitivity and specificity for the detection of infection by high-risk serotypes of HPV and, in the appropriate clinical context, bowenoid papulosis [5]. Given reports of the transformation of bowenoid papulosis into invasive squamous cell carcinoma, continued clinical monitoring is recommended [6]. However, a previous study has shown that, compared to areas such as the cervix and anogenital region, high-risk HPV is rarely detected in extra-anogenital cutaneous squamous cell carcinoma [7].

A major concern in bowenoid papulosis of the head and neck region is the potential for oropharyngeal involvement with HPV-induced squamous cell carcinoma. ENT evaluation should be considered on a case-by-case basis with extragenital bowenoid papulosis of the head and neck due to the proximity to the oropharynx and the potential for squamous cell carcinoma. Suspicious symptoms warranting further scrutiny include dysphagia, dysphonia, odynophagia, otalgia, nonhealing mouth ulcers, and bleeding. In addition, given the possibility of a subclinical lesion in the genital or extragenital sites, we advocate for a periodic follow-up to detect new lesions or possible progression to SCC. While our patient denied a history of oral sex, previous reports have suggested the possibility of viral spreading through shared razors or other hygiene products [8].

Interestingly, several cases of extragenital bowenoid papulosis have been reported in immunosuppressed patients. This includes two cases in patients with HIV and one with CD4+ lymphocytopenia [2,4,9]. Together, these reports suggest that immunosuppression may play a role in the development of this rare condition. Further, immunosuppression may increase the risk of the development of squamous cell carcinoma in these cases, as has been seen with classical bowenoid papulosis [6].

## Conclusions

Our case demonstrates that extragenital bowenoid papulosis should be considered in unusually appearing, isolated plaques of the head and neck, particularly in an immunosuppressed patient. Further, additional testing for high-risk HPV should be pursued whenever bowenoid features are observed on biopsy. In addition, ENT consultation should be considered on a case-by-case basis for those patients with a positive review of symptoms where oropharyngeal involvement and invasive squamous cell carcinoma are possibilities.
